# The effect of increased channel interaction on speech perception with cochlear implants

**DOI:** 10.1038/s41598-021-89932-8

**Published:** 2021-05-17

**Authors:** Tobias Goehring, Alan W. Archer-Boyd, Julie G. Arenberg, Robert P. Carlyon

**Affiliations:** 1 Cambridge Hearing Group, MRC Cognition and Brain Sciences Unit, University of Cambridge, 15 Chaucer Road, Cambridge, CB2 7EF UK; 2 Massachusetts Eye and Ear, Harvard Medical School, 243 Charles St, Boston, MA 02114 USA

**Keywords:** Cognitive neuroscience, Peripheral nervous system, Sensory processing, Medical research, Biomedical engineering

## Abstract

Cochlear implants (CIs) are neuroprostheses that partially restore hearing for people with severe-to-profound hearing loss. While CIs can provide good speech perception in quiet listening situations for many, they fail to do so in environments with interfering sounds for most listeners. Previous research suggests that this is due to detrimental interaction effects between CI electrode channels, limiting their function to convey frequency-specific information, but evidence is still scarce. In this study, an experimental manipulation called spectral blurring was used to increase channel interaction in CI listeners using Advanced Bionics devices with HiFocus 1J and MS electrode arrays to directly investigate its causal effect on speech perception. Instead of using a single electrode per channel as in standard CI processing, spectral blurring used up to 6 electrodes per channel simultaneously to increase the overlap between adjacent frequency channels as would occur in cases with severe channel interaction. Results demonstrated that this manipulation significantly degraded CI speech perception in quiet by 15% and speech reception thresholds in babble noise by 5 dB when all channels were blurred by a factor of 6. Importantly, when channel interaction was increased just on a subset of electrodes, speech scores were mostly unaffected and were only significantly degraded when the 5 most apical channels were blurred. These apical channels convey information up to 1 kHz at the apical end of the electrode array and are typically located at angular insertion depths of about 250 up to 500°. These results confirm and extend earlier findings indicating that CI speech perception may not benefit from deactivating individual channels along the array and that efforts should instead be directed towards reducing channel interaction per se and in particular for the most-apical electrodes. Hereby, causal methods such as spectral blurring could be used in future research to control channel interaction effects within listeners for evaluating compensation strategies.

## Introduction

Cochlear implants (CIs) are surgically implanted hearing devices that provide a sense of sound for people with severe-to-profound hearing loss. They do so by replacing the function of damaged sensory hair cells, responsible for the acoustic-to-neural transduction process of sound, with electrical stimulation through an array of electrode contacts placed inside the cochlea of the recipient. CIs are successful in providing speech perception benefits for most recipients in quiet acoustic situations. However, CI recipients experience speech perception difficulties in situations with background noise^1,2^ and there is large variability in outcomes between recipients^3^. The reasons for the limitations and variability in outcomes are not fully understood and it remains unclear why some interventions (e.g. novel sound coding strategies^4–6^, current focussing techniques^6,7^ or noise reduction algorithms^8,9^) provide benefits for some listeners and acoustic situations, but harm speech perception in others. One main reason for the perceptual difficulties in noisy situations is the large spread of neural activation along the auditory nerve (*spread of excitation*) with the monopolar stimulation mode used in clinical CI devices. The resulting overlap in neural excitation between adjacent electrode channels leads to degradations of the spectro-temporal resolution and is thought to reduce the number of independent channels of information that can be utilized for speech recognition^1,10,11^.

Numerous attempts have been developed to overcome this limitation and to reduce the detrimental effects of spread of excitation and channel interaction with CIs. Several studies used current focusing strategies based on multi-polar stimulation to restrict the spread of excitation and to reduce interaction between adjacent electrode channels^6,7,12–16^. So far, results have been mixed, and only one study found benefits for CI speech perception for most recipients by using current focusing^7^. Other studies proposed the stimulation of only a subset of the intracochlear electrodes, which were identified based on electrode-wise measurements of perceptual or physiological parameters. The restricted use of a selection of the best electrode channels was predicted to produce good speech perception while reducing the channel interaction and interference due to the least effective electrode channels^6,17–22^. Again, speech perception results have been mixed. Furthermore, many different measures have been used to select the deactivated electrodes, and it has recently been shown that they result in different selections of electrodes for deactivation^23^; it remains elusive how these measures interact and how to identify the best subset of electrodes on an individual basis. It is therefore important to address the limited understanding of how the effects of spread of excitation and channel interaction affect speech perception in noise with CIs.

Channel interaction effects have traditionally been investigated in normal-hearing individuals listening to vocoded speech in an attempt to simulate the information conveyed by cochlear implant processing^10,24–29^. These simulation studies revealed that a spectral filter slope of about − 12 dB / octave and a total number of about 4–8 channels leads to similar speech performance for normal-hearing as occurs in CI hearing. However, the simulations suffered from several limitations and do not fully account for the electrical stimulation, neural excitation and resulting channel interaction effects with CIs. Recently, we used a manipulation of spread of excitation directly in CI listeners to investigate channel interaction effects on speech perception in noise^30^. To do so we applied a manipulation called *spectral blurring* in two experiments: one that used overlapping bandwidths of the CI analysis filters and one that used simultaneous stimulation of adjacent electrode contacts for each frequency channel to widen the spread of excitation and to investigate the causal effects of channel interaction in CIs. In both experiments, the channel interaction due to spectral blurring led to increased difficulties in speech perception in noise by CI listeners when all electrode channels were affected. However, spectral blurring did not affect speech-in-noise perception when only a subset of 5 evenly-spaced electrodes were affected, and also not when these 5 electrodes were deactivated. This latter finding indicated that deactivation of electrode channels may not provide benefits in speech perception at least when the electrode channels are spaced evenly along the array. However, these findings were limited to the particular speech test, that used a competing talker as background noise, and it remains unknown if speech perception would have been affected in other types of noise or even in quiet without any background noise present. Surprisingly, spectral blurring did not affect speech perception for the specific case when 5 evenly-spaced electrodes were blurred, but this could potentially be explained by the large redundancy of speech information across the frequency spectrum and the wide filter bandwidths used in CIs, both of which lead to strong correlations between adjacent electrode channels. The CI listeners may have been able to ignore the blurred electrode channels and to receive that information from adjacent, non-blurred electrode channels.

In this study, spectral blurring was used to further investigate the causal relationship of channel interaction with CI speech perception in noise and in quiet. The first part investigated whether speech perception is degraded by spectral blurring in situations without any background noise and when a more realistic multi-talker noise is used. The comparison of these two scenarios informs about whether the manipulated channel interaction due to spectral blurring predominantly increased the masking of speech information by background noise, or if the increased channel interaction leads to speech distortions that directly impact speech perception per se. The second part investigated the effects of spectral blurring on speech perception in quiet and in noise when groups of adjacent, clustered electrodes were blurred in comparison to when only evenly-spaced electrodes were blurred. Due to the fixed positioning of the electrodes within the CI electrode array, it seems more likely that clusters of electrodes, rather than individual electrodes, would be ill-positioned in relation to the auditory nerve. Speech performance with spectrally blurred clusters of 5 electrodes for basal, middle and apical regions was compared to 5 evenly-spaced electrodes along the array to assess the effect of clustering and of frequency/location-dependent effects.

## Results

**Figure 1:**
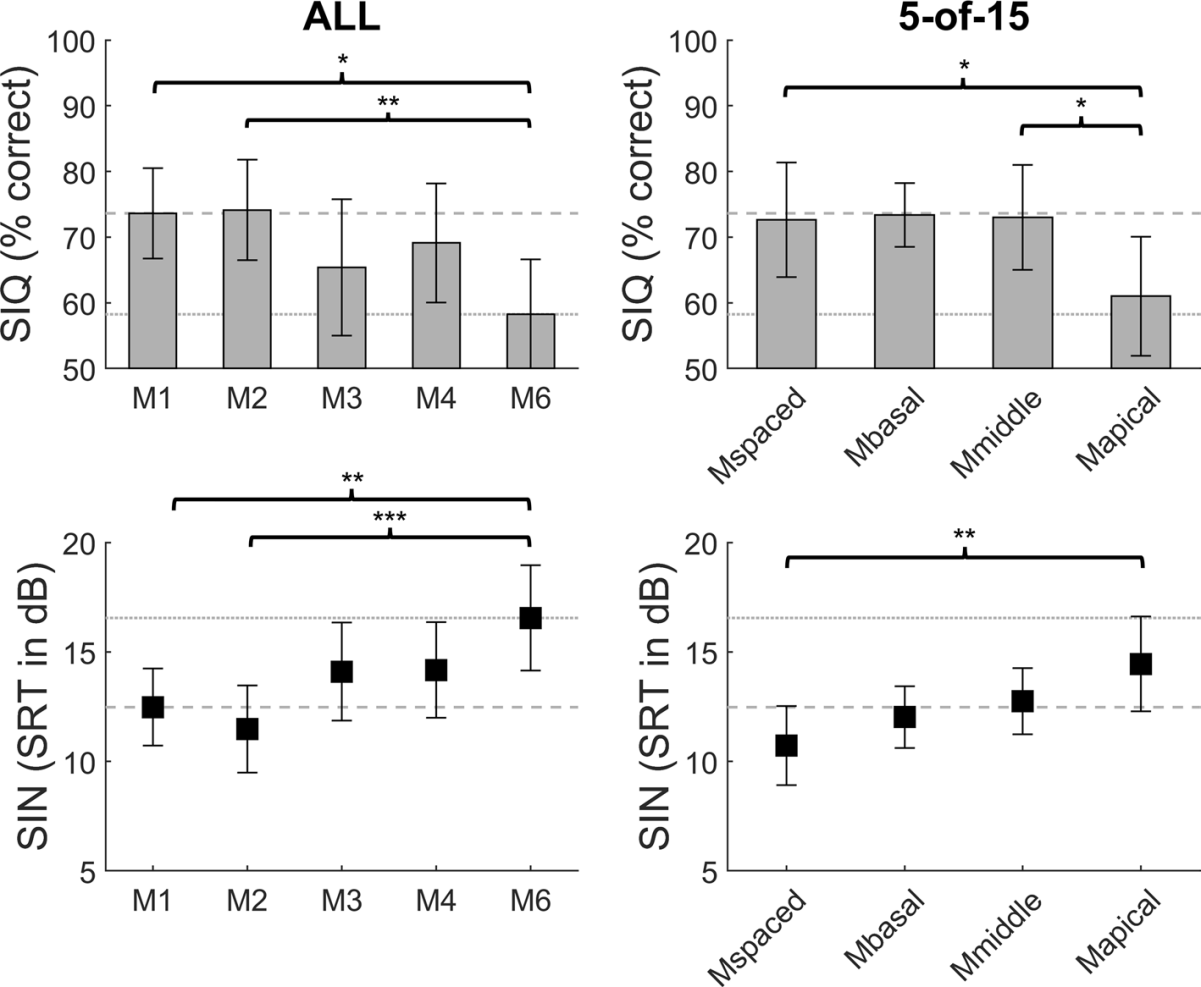
Speech perception results as mean scores for the group of 8 CI recipients when using spectral blurring for all 15 electrode channels (ALL, left) and for 5 out of 15 electrode channels (5-of-15, right). Top: speech perception in quiet (SIQ, in percentage correct), Bottom: speech reception threshold in multi-talker noise (SIN, in dB SNR). Note that for the ALL condition, the map number indicates the blurring factor and in the 5-of-15 condition, all maps used a blurring factor of 6. Error bars indicate standard errors and the dashed and dotted lines were added for visual comparison between the ALL and the 5-of-15 conditions (*: *p* < 0.05; **: *p* < 0.01; ***: *p* < 0.001).

The speech perception results for the group of CI listeners are shown in Fig. 1.

The first experiment evaluated the effect of spectral blurring applied to all 15 active electrode channels (ALL, Fig. 1 left). Spectral blurring in the ALL condition was found to degrade speech perception by up to 15.9% for speech in quiet (SIQ) and by up to 4.1 dB SRT for speech in multi-talker noise (SIN). For speech in quiet, statistical analysis using a linear mixed model revealed a significant effect of spectral blurring for ALL on speech perception scores (*F*(4,32) = 4.52, *p* = 0.005) and pairwise comparisons using Tukey's HSD tests indicated that there were significant differences between M1 (no blurring) and M6 (six-fold blurring; − 15.4%, *t*(32) = 3.24, *p* = 0.014) and between M2 and M6 (− 15.9%, *t*(32) = 3.57, *p* = 0.009). For speech in noise, statistical analysis revealed a significant effect of spectral blurring for ALL on speech perception scores (*F*(4,32) = 7.78, *p* < 0.001) and pairwise comparisons using Tukey's HSD tests indicated that there were significant differences between M1 and M6 (4.1 dB SRT, *t*(32) = − 4.16 *p* = 0.002) and between M2 and M6 (5.1 dB SRT, *t*(32) =  − 5.18, *p* < 0.001). These differences for SIQ (15%) and for SIN (4–5 dB SRT) are considered to be clinically significant and show that spectral blurring with a factor of 6 degraded CI speech perception. After normalizing each subject’s score by subtracting their average performance across maps, speech scores in quiet and in noise were significantly correlated (*r* = − 0.53, *p* = 0.002), suggesting that the effects of blurring were consistent across subjects between speech tests. Furthermore, the effect sizes of blurring were similar between SIQ (Cohen’s *d* = 0.71) and SIN (*d* = 0.69) for the differences between M1 and M6.

The second experiment evaluated the effect of spectral blurring applied to a subset of 5 out of 15 active electrode channels (5-of-15, Fig. 1 right) that were either clustered or evenly-spaced along the cochlea. In these conditions a blurring factor of 6 was applied to the selected electrodes. For speech in quiet (SIQ), spectrally blurring 5 evenly spaced channels produced similar scores to that obtained with no blurring at all, as indicated by the dashed grey line extending from the M1 condition in the left-hand plot. This was also true when blurring was applied to 5 adjacent electrodes in the basal and middle parts of the array. However, blurring 5 apical electrodes decreased performance by 12%, to a level roughly similar to that when all electrodes were blurred („M6 “, dotted grey line in Fig. 1). Statistical analysis revealed a significant effect of which 5 channels were blurred (*F*(3,24) = 4.39, *p* = 0.013) and pairwise comparisons using Tukey's HSD tests indicated that there were significant differences between Mspaced and Mapical (− 11.6%, *t*(24) = 3.16, *p* = 0.021) and between Mmiddle and Mapical (− 12%, *t*(24) = 2.97, *p* = 0.032). For speech in noise, blurring 5 equally spaced electrodes did not increase SRTs relative to the case with no blurring (“M1”, dashed grey line in Fig. 1). This was also true for blurring 5 adjacent electrodes in the basal or middle part of the array. Consistent with the results obtained in quiet, blurring five adjacent apical channels did decrease performance (increase SRTs) in noise. Statistical analysis revealed a significant effect of which 5 channels were blurred (*F*(3,24) = 5.46, *p* = 0.005); pairwise comparisons using Tukey's HSD tests indicated that there was a significant difference between Mspaced and Mapical (3.7 dB SRT, *t*(24) = − 3.96, *p* = 0.003). After normalizing each subject’s score by subtracting their average performance across maps, speech scores in quiet and in noise were significantly correlated (*r* = − 0.68, *p* < 0.001), suggesting that the effects of blurring were consistent across subjects between speech tests. Hereby, the effect size of blurring in SIQ (*d* = 0.46) was somewhat smaller than for SIN (*d* = 0.68) for the differences between Mspaced and Mapical, but can be considered similar still.

## Discussion

As expected from previous findings, applying spectral blurring to all channels of a CI impaired speech perception in noise, but this was also true for speech in quiet. When the largest, six-fold, blurring was applied, speech perception scores dropped by 15% in quiet, and SRTs increased by 4 dB in multi-talker noise, relative to a condition with no blurring. The effects of blurring were consistent between the two speech tests administered, with medium-to-large effect sizes for speech perception in quiet and in noise (Cohen’s d of 0.69 and 0.71). We would have expected that the effect of spectral blurring is somewhat exacerbated in background noise, due to the summation of the detrimental effects of increased masking by noise *and* distorted speech information. However, the SIQ test, which was always administered first, may have provided some acclimatization to blurring and reduced its effect in the following SIN test. The marked difference in speech scores between the baseline (unblurred) condition and that when all channels were strongly blurred suggests that, at least when channel interactions are unusually large, speech perception may be substantially improved by strategies that reduce channel interactions effectively, such as for example *effective* focused stimulation.

The most important finding arose when we applied spectral blurring to only a subset of electrodes along the array. As mentioned in the Introduction, the idea of deactivating electrodes that produce detrimental channel interaction is presently of great scientific and clinical interest. We found that, in many conditions, applying even extreme (six-fold) blurring to 5 out of the 15 electrodes did not impair the perception of sentences either in quiet or in the presence of multi-talker noise. This was true when the blurred electrodes were evenly spread across the array (as shown previously for speech in a single-talker noise^30^), but also when they occupied 5 adjacent positions in either the basal or middle turn of the array. All of these conditions produced statistically indistinguishable performance, and the only condition that was substantially worse was when the five most apical electrodes were blurred. This led to medium-to-large effect sizes in quiet (d = 0.46) and in noise (d = 0.68) when comparing to the map that used evenly-spaced blurring. The clinical importance is clear: deactivating electrodes solely because they produce blurred excitation patterns is unlikely to help, unless the deactivation is applied to electrodes next to each other and at the apical end of the array. We base this conclusion on the assumption that, if a blurred representation does not impair speech perception, then correcting it by deactivating that electrode is unlikely to improve matters. The scientific importance, discussed below, concerns the role of low-frequency information, conveyed to the most apical region of the cochlea that is stimulated by the electrodes, on speech perception.

The five most apical channels in our listeners’ CIs convey frequency information up to about 1 kHz and are typically located at angular insertion depths from about 250° to 500°^31^. This frequency region spans important spectral features of speech including the first formants (up to about 1000 Hz). Access to this low-frequency information is especially important for speech perception with CIs^32,33^, in that electrode discrimination ability in the low-to-medium frequencies (up to 2700 Hz) is more difficult and correlated with speech perception for CIs^32^, that band-importance functions rely more heavily on lower frequencies (200 – 400 Hz) with CIs^33^ and that apical *holes in hearing* affected speech perception with CIs strongest^34^. The latter study by Shannon et al. simulated *holes in hearing* by deactivating, or reassigning, adjacent channels in either the basal, middle or apical region of the electrode array with 20-channel Cochlear devices and found that their manipulation did not degrade speech perception when applied to the basal region, but when applied to the middle and even more so when applied to the apical region. While they used a different manipulation (holes, rather than increased channel interaction), their findings correspond to our findings with blurring (both used holes/blurred clusters spanning about 6 mm) in that the apical electrode channels are most important for speech perception. This aligns with an early study reporting that the most-apical electrode location had the strongest influence on vowel recognition with CIs^35^, even though settings differed from current CIs. Further evidence potentially comes from studies that found greater insertion depths to be associated with better speech perception outcomes with CIs, possibly by reaching more apical spiral ganglion neurons^3,36,37^, but two studies on participants using the HiFocus 1J and MS electrodes found that performance was not correlated with position-related variables when controlling for patient-specific predictors such as pre-implant speech scores and duration of deafness^31,38^. For both MS and 1J electrodes used here, average distances from the inner wall of the cochlea were smallest for electrodes 1–5, potentially exacerbating the effect of blurring in the apical region over the other regions that already suffer from larger current spread due to larger electrode-neuron distances^31^. However, the negative effect for the apical cluster of blurred channels needs to be confirmed for other electrode types, before conclusions can be extrapolated to other CI devices with different insertion depths and/or electrode spacings. More generally, provision of concurrent low-frequency information via electric-acoustic^39–41^ or electric-tactile stimulation has been shown to enhance speech-in-noise perception with CIs^4,42–44^. Although such low-frequency information may be unintelligible on its own, and may consist simply of an amplitude-modulated tone, it has been shown to provide phonetic cues relevant to voicing and glimpsing to benefit speech listening^42,45,46^. For acoustic hearing, a speech intelligibility model still reached close-to-maximum prediction performance when restricted to low-frequency information^47^ in the range of 100 to 1000 Hz. Furthermore, low-frequency information up to 1000 Hz was found to provide vital cues for acoustic landmarks that facilitate listening in noise^48^. All of these aspects are likely to contribute to the location-specific effect of spectral blurring along the cochlea, with a significant degradation of speech perception when applied to the five most apical electrodes.

In conclusion, this study provides further evidence for the widely-supported assumption that speech perception with cochlear implants is strongly affected by channel interaction due to overlap between adjacent frequency channels. The novel experimental manipulation used, that applied spectral blurring as a simulation of increased channel interaction directly in CI recipients, demonstrated the causal effect of this fundamental mechanism in cochlear implant listening. The within-participant design allowed us to isolate the effect of channel interaction to evaluate its impact on speech perception. Importantly, it was found that spectral blurring only affected speech perception when applied to all electrodes, or to a cluster of one third of the electrodes in the apical region of the cochlea spanning frequencies up to about 1 kHz. Blurring applied to a subset of evenly-spaced electrodes or to electrodes clustered in other parts of the array produced performance that did not differ significantly from each other and that was not worse than when no blurring was applied. This provides further evidence for the assumption that CI listening is dependent on effective transmission of low-frequency speech information, even in situations without background noise. These findings could have important consequences for the development of future strategies that aim to improve speech perception with CIs, by indicating that interventions targeting more apical regions may be more likely to succeed whereas strategies that target a set of evenly spaced electrode channels seem less likely to succeed. These results were obtained using sentences as speech stimuli that may allow for some top-down compensation of the blurred representation based on context. Future research should evaluate whether the effects of spectral blurring hold also for other types of speech tests such as phoneme or vowel identification, for instance. Furthermore, research studies could use within-subject manipulations such as spectral blurring to avoid confounding, between-subject factors related to cognitive or language skills for the preliminary evaluation of novel CI processing strategies in controlled settings. For example, spectral blurring could be used to test future CI coding strategies that aim to reduce channel interaction to see whether these strategies can compensate for the detrimental effect of blurring on speech perception.

## Methods

### Participants

A group of 8 adult CI users (4 female), all native English speakers and unilaterally implanted with an Advanced Bionics HR90K CI and HiFocus 1J or MS electrode, participated in this research. The 1J electrode for outer-wall positioning has 16 contacts extending over a length of 17 mm with a spacing of 1.1 mm and an average insertion depth of 478° (SD = 66)^31^. The pre-curved MS electrode has 16 contacts extending over a length of 15 mm with a spacing of 0.9 mm and an average insertion depth of 424° (SD = 29). Most participants used the Optima-S strategy, one used the Fidelity 120 strategy, and all had more than 3 years of listening experience with their CI. All participants had good or very good clinical speech performance (> 75% percent correct). The mean age of the group was 66.6 years, ranging from 49 to 75 years. Only the implanted ear was used for the testing and stimuli were presented via direct connection (auxiliary input) to a research processor worn by the participants. If the participant had a hearing aid in the other ear, then it was taken off during the test to minimize distractions. Prior to the testing session, the participant's clinical CI program parameters were obtained to define the initial settings for the research processor settings and to exclude any electrodes from the testing that were deactivated in their clinical settings. All eight participants were also part of an earlier study that investigated spectral blurring^30^, but were blinded as to what type of manipulation they were listening to. Participant demographics together with details of the clinical CI processing strategy are shown in Table 1.

**Table 1:** Participant demographic information and clinical CI settings.

Participant	Sex	Age (y)	Duration implanted (y)	Duration of deafness (y)	CI speech processor, Implant	CI electrode array	Clinical strategy, pulse width (us)	Electrodes deactivated in clinical MAP
AB24	F	49	3	4	Naida CI Q90, HR90K Advantage	HiFocus MS	HiRes Optima-S, 35	15, 16
AB1	M	74	10	41	Naida CI Q90, HR90K	HiFocus 1J	HiRes Optima-S, 26	16
AB20	M	73	3	40	Naida CI Q90, HR90K Advantage	HiFocus MS	HiRes Optima-S, 29.6	–
AB3	M	72	11	36	Naida CI Q90, HR90K	HiFocus 1J	HiRes Optima-S, 29.6	–
AB6	F	70	5	65	Naida CI Q90, HR90K	HiFocus 1J	HiRes Optima-S, 35	16
AB2	F	60	11	27	Naida CI Q90, HR90K	HiFocus 1J	HiRes Optima-S, 31.4	16
AB23	F	60	3	58	Naida CI Q90, HR90K Advantage	HiFocus MS	HiRes Optima-S, 23.3	–
AB19	M	75	3	n.a	Naida CI Q90, HR90K Advantage	HiFocus MS	HiRes Fidelity 120, 18	–

Ethical approval was obtained from the National Research Ethics committee for the East of England before commencing the research. All research was performed in accordance with the relevant guidelines and regulations. Participants gave their informed consent and were paid for partaking as well as reimbursed for travel expenses.

### Processing conditions based on spectral blurring

Channel interaction effects between adjacent CI electrode channels arise from the large overlap in neural excitation with monopolar stimulation, in particular for electrodes located further away from the targeted spiral ganglion neurons. This study used a direct manipulation of channel interaction in CI recipients in contrast to previous studies that used normal hearing listeners and CI vocoder simulations.

This manipulation simulated increased channel interaction effects due to wide current spread in CIs by simultaneously stimulating several adjacent electrodes for a given electrode channel. In the standard clinical program used by Advanced Bionics CI devices, either a single electrode (HiRes) or a weighted combination of two electrodes ("current steering" in HiRes Fidelity 120 or Optima strategies) are used for conveying the information of each filter bank channel. The individual filter bank channels are constructed by summing the outputs of a Fast Fourier Transform (FFT) with 256 bins (with a bandwidth of 64 Hz per bin) with increasing numbers of bins for increasing center frequencies to align with human frequency selectivity. Due to the use of a BlackHann window (average of Blackmann and Hanning), the sidelobe attenuation of the FFT processing is about 43 dB and rolling off with 18 dB per octave. This means that while there is considerable sideband attenuation, some acoustic energy will spill over into adjacent filter bank channels and reduce the frequency selectivity of the filter bank leading to some spectral blurring (in fact this is what we manipulated with *input blurring* previously^30^). However, this affected every map used in this experiment to a similar degree due to the use of the same filter bank for all maps, and should therefore not lead to differences in performance between maps.

**Figure 2:**
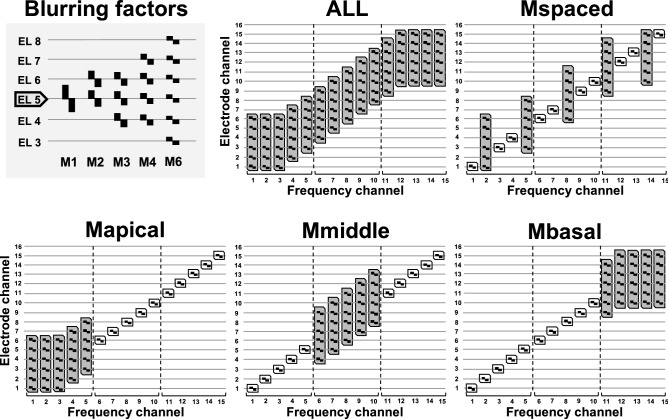
Illustration of the electrode stimulation pattern used for spectral blurring. The top left part shows the blurring factors of 1 to 6 simultaneous electrodes used in the experimental maps (centred at electrode 5 in this example). The ALL plot shows a condition using 6 simultaneous stimulation electrodes for each of the 15 active frequency channels. The 5-of-15 conditions (Mspaced, Mapical, Mmiddle and Mbasal) use clusters of 5 adjacent or 5 evenly-spaced electrodes with a blurring factor of 6 (grey).

This experiment used spectral blurring at the electrode level to simulate the effects of increased channel interaction by adjusting the number of simultaneously stimulated electrodes for each *blurred* filter bank channel^30^. Electrode channels were blurred with 4 different blurring factors: 2, 3, 4 or 6 simultaneously stimulated electrodes each using the same stimulation current. Two different blurring conditions were used in this experiment, one that applied spectral blurring to all 15 electrodes ("ALL") and one that blurred only a subset of 5 out of 15 electrodes ("5-of-15"). Note that the most basal electrode 16 was deactivated for all subjects and maps, so as to make the experimental maps more similar across participants. In the ALL condition, 5 different experimental MAPs were generated according to the blurring factor used (M1, M2, M3, M4, M6 for blurring factors, or simultaneously-stimulated electrodes per filter channel, of 1, 2, 3, 4, 6, respectively). Note that in the ALL condition, the number of the MAP is given by the blurring factor used. For the 5-of-15 condition, 4 different experimental MAPs were generated all of which used a blurring factor of 6, that was applied to 5 equally-spaced electrodes (electrodes 2, 5, 8, 11, 14, "Mspaced") or to 5 adjacent electrodes in the apical (electrodes 1–5, "Mapical"), the middle (electrodes 6–10, "Mmiddle") or the basal (electrodes 11–15, "Mbasal") region of the electrode array. For the MS electrode, the blurred electrodes were located in cochlea regions corresponding to average angular insertion depths of 40° to 130° (Mbasal), 140° to 230° (Mmiddle) and 250° to 425° (Mapical). For the 1J electrode, these regions were shifted by about 50° apically on average due to the distance in average insertion depths. The 5 adjacent electrodes in the three maps using clustered blurring conveyed frequency information from 2141 – 8054 Hz, 918 – 2141 Hz and 238 – 918 Hz, respectively. In total there were 9 different test conditions, five in the ALL condition and four in the 5-of-15 condition. Figure 2 shows an illustration of the electrode patterns used for spectral blurring.

### Stimuli and speech tests

Each of the processing conditions were evaluated using two speech perception experiments. The first part measured the perception of speech in quiet (SIQ), without background noise. This part used the IEEE speech corpus^49^, spoken by a British male talker, which consists of 72 lists with 10 sentences with 5 keywords each. Speech perception was assessed for SIQ as the percentage of keywords identified correctly for 2 lists presented per condition (20 sentences). Lists were chosen randomly without replacement.

The second part measured the perception of speech in noise (SIN). In contrast to the previous part, the SIN part used the BKB speech corpus^50^ in combination with the 20-talker babble noise from Auditec (St. Louis). The BKB sentences, used clinically in the UK, are typically easier than the IEEE sentences and were used to assess performance with a different speech corpus in addition to the IEEE sentences. The BKB lists consist of 15 sentences with 3 keywords each, spoken by a British male talker. The SIN test procedure^51^ used an adaptive one-up/one-down procedure to measure the speech reception threshold (SRT) at which 50% of the sentences were understood correctly. Starting with an initial signal-to-noise ratio (SNR) of 4 dB SNR, the procedure used a randomly selected sentence and a stepsize of 2 dB per trial to alter the noise level until the participant repeated the three keywords of the first sentence correctly. It then started with the adaptive one-up/one-down procedure to determine the SRT for that list as the average of the last ten SNRs presented. Two lists of 15 sentences were presented for each condition and the final SRT score was calculated as the average of the two SRTs.

During the experiment, participants took off their own clinical CI speech processor and used an Advanced Bionics research speech processor connected with an auxiliary cable to an external sound card (Roland Quad-Capture) and laptop computer (Dell XPS 15). The fitting procedure for the research speech processor parameters and the programming of the experimental MAPs onto the research speech processor was performed using BEPS + software from Advanced Bionics. The research software for the speech tests was programmed in MATLAB (The Mathworks).

### Experimental procedure

The experiment was split into two 3-h sessions per participant. In the first session, the settings of the experimental MAPs were determined using a loudness fitting procedure for all electrodes and all MAPs. The fitting procedure always started with M1, for which all threshold (T) and most-comfortable (M) levels were obtained by increasing the stimulation current for each electrode individually until the participant confirmed the level and using the BEPS + software by Advanced Bionics. Hereby, a loudness rating chart was used that contained 10 loudness levels from 1 "Just noticeable" up to 10 "Too loud", and from which the first level was used as T-level and the sixth level "Comfortable" was used as M-level. After the electrode-wise T- and M-levels were obtained, all electrodes were played in direct succession at M-level to allow for a comparison between electrodes. If the participant indicated that the loudness for the current electrode was notably different from the previous electrode, then this was corrected by changing the stimulation level of the current electrode until the participant indicated a comparable loudness to the adjacent electrodes. All other experimental MAPs were based on M1, which ensured that the same processing settings were used across MAPs (ie. using the pulse width and rate of the clinical settings for each participant). All T- and M-levels were fitted for each experimental MAP using this procedure, and any non-linear front-end processing (such as noise reduction or automatic gain control) was deactivated, maintaining identical settings across MAPs. In the second session, the speech tests were firstly performed for the five ALL conditions (M1, M2, M3, M4 and M6) and secondly for the four 5-of-15 conditions (Mspaced, Mbasal, Mmiddle and Mapical). For both conditions, the experimental maps were quasi-counterbalanced across participants. The speech was presented at a comfortable listening level which was set using the volume control of the soundcard prior to starting the speech test for each participant and experimental MAP. For both testing sessions, which were performed within 2 weeks, participants were offered short breaks throughout the testing and each session lasted about 2–3 h. Participants were blinded as to which condition they were listening to.

Results were analysed using R statistical software (RStudio Version 1.3.959; packages: lmerTest, lme4, emmeans). Linear mixed-effects models were fitted to the results data with speech score as outcome measure, blurring condition as fixed factor and participant as random factor. P-values for the fixed-effects term were calculated using F tests (Satterthwaite’s approximation of degrees of freedom). Post-hoc tests were performed for pairwise comparisons between all conditions in a given test using Tukey's HSD test to correct for multiple comparisons using the emmeans library. Note that for the statistical analysis, percentage correct scores in the SIQ condition were transformed using the rationalized arcsine transform^52^ (RAU).
